# Family-based psychosocial interventions for adult Latino patients with cancer and their caregivers: A systematic review

**DOI:** 10.3389/fpsyg.2023.1052229

**Published:** 2023-03-30

**Authors:** Ting Guan, Paz Cook, Shenmeng Xu, Lisa Hart Ranzinger, Jamie L. Conklin, Abdulrahman Abdulmuslih S. Alfahad, Yu Ping, Karl Shieh, Susana Barroso, Natalia Villegas, Lixin Song

**Affiliations:** ^1^School of Social Work, Syracuse University, Syracuse, NY, United States; ^2^Gillings School of Public Health, University of North Carolina at Chapel Hill (UNC), Chapel Hill, NC, United States; ^3^School of Nursing, University of North Carolina at Chapel Hill (UNC), Chapel Hill, NC, United States; ^4^Jean and Alexander Heard Libraries, Vanderbilt University, Nashville, TN, United States; ^5^Health Sciences Library, University of North Carolina at Chapel Hill (UNC), Chapel Hill, NC, United States; ^6^Jacob School of Engineering, University of California at San Diego, San Diego, CA, United States; ^7^School of Nursing, University of Texas Health San Antonio, San Antonio, TX, United States

**Keywords:** cancer, oncology, family-based psychosocial intervention, caregiver, quality of life, Latino/Latina/Latinos, systematic review

## Abstract

**Objective:**

This review aimed to systematically examine the characteristics and outcomes of family-based psychosocial interventions offered to adult Latino patients with cancer and their caregivers.

**Methods:**

We searched six databases from their inception dates through June 2022. Studies were eligible for inclusion if they (1) targeted both adult Latino patients diagnosed with cancer and their adult caregivers or reported subgroup analyses of Latino patients and caregivers; (2) included family-based psychosocial interventions; (3) used randomized controlled trial (RCT) or quasi-experimental designs; and (4) were published in English, Spanish or Portuguese. Members of our multidisciplinary team assessed the risk of bias in the reviewed studies using the Cochrane Collaboration's Risk of Bias Tool.

**Results:**

Our database searches yielded five studies. The studies were conducted in the U.S. and Brazil. Three studies were RCTs, and two used quasi-experimental designs. The sample sizes ranged from 18 to 230 patient-caregiver dyads. These studies culturally adapted the intervention contents and implementation methods and involved bilingual interventionists. The interventions had beneficial effects on multiple aspects of psychosocial outcomes for both patients and caregivers. We also identified methodological limitations in the reviewed studies.

**Conclusions:**

Findings from this systematic review help deepen our understanding of family-based psychosocial interventions for Latinos affected by cancer. The small number of psychosocial interventions focused on adult Latino cancer patients and their caregivers is concerning, considering that Latino populations are disproportionally burdened by cancer. Future research needs to design and evaluate culturally-appropriate interventions to support Latino patients and families who cope with cancer.

**Systematic review registration:**

https://www.crd.york.ac.uk/prospero/display_record.php?RecordID=274993, identifier CRD42021274993.

## 1. Introduction

The Hispanic/Latino population in the United States (U.S.; referred to as “Latinos” henceforth) constitutes the largest and fastest-growing racial and ethnic minority group in the country (American Cancer Society, 2021). With an estimated amount of 176,600 new cancer cases and 46,500 cancer deaths in the U.S in 2021 (Miller et al., 2021), Latino individuals with cancer experience higher levels of symptoms, psychological distress (Alcalá, 2014), poorer health-related quality of life (Nahleh, 2016), and greater unmet psychosocial needs (Moadel et al., 2007) than other populations in the U.S. The disparities in cancer outcomes have been associated with social determinants of health. Latinos in the U.S. have lower socioeconomic status than non-Hispanic Whites. Data from U.S. Census Bureau shows that in 2019, the poverty rate was 16% for Latinos as compared to the poverty rate of 7% for non-Hispanic Whites (Creamer, 2020). In addition, language barriers contribute to health care disparities for Latinos in the United States of America. Latinos also comprise the highest percentage of people without health insurance in the U.S (American Cancer Society, 2021). As a result, they have less access to health care and psychosocial services (i.e., social work and psychological services; Costas-Muñiz et al., 2017), especially culturally and linguistically competent care.

Family caregivers, referring to unpaid individuals identified by the Latino patients with cancer as the primary caregiver(s) who are involved in providing direct assistance and/or support to the patient (e.g., family members, friends). One of the central cultural values for Latinos is familismo or familisism, which highly emphasizes the importance of family loyalty, support, connections, and interdependence (Valdivieso-Mora et al., 2016). Previous research has demonstrated that Latino caregivers play essential roles in providing instrumental and emotional support (National Alliance for Caregiving, 2019), decision making (Shen et al., 2020), and behavioral changes across the cancer survivorship trajectory (Skiba et al., 2022). However, Latino caregivers experience numerous challenges, such as communication difficulties with health providers (Wells et al., 2008), social isolation (King et al., 2022), and difficulties in finding respite care to give them relief (National Alliance for Caregiving, 2019). Latino caregivers often report more burden regarding to finances and lack of family support, and poorer mental health compared to non-Hispanic White caregivers (Siefert et al., 2008).

A growing body of research suggests that family-based psychosocial interventions hold great potential that can benefit both patients and their family caregivers in psychological functioning, marital functioning, and quality of life (Northouse et al., 2010). However, little is known about the developing science regarding interventions that address the survivorship care needs of Latino patients with cancer and their family caregivers (McNulty et al., 2016). Given the strong family allegiance and attachment in Latino culture and their living context regarding to social determinants of health, this systematic review aimed to examine the characteristics of family-based psychosocial interventions offered to adult Latino patients with cancer and their caregivers and assess the intervention outcomes.

## 2. Methods

This review has been registered on the PROSPERO, the International Prospective Register of Systematic Reviews (Registration number CRD42021274993).

### 2.1. Eligibility criteria

The studies were included in the review if they met the following inclusion criteria: (1) targeted both adult Latino patients (18 years of age) diagnosed with cancer and their adult caregivers (i.e., family members, close friends, or anyone identified by patients as the primary supportive person involved in providing direct assistance to the patient) or included subgroup analyses of Latino participants; (2) included interventions that were family-based (defined as an intervention targeted both patients and their family caregivers or an intervention targeted one member of the dyad to improve the outcomes of both the patients and their caregivers) and had psychosocial or behavioral components; (3) used a randomized controlled trial or a quasi-experimental design; and (4) were published in English, Spanish, or Portuguese.

### 2.2. Search strategy

We searched the following databases from their dates of inception through June 2022: PubMed, CINAHL Plus with Full Text (EBSCOhost), APA PsycInfo (EBSCOhost), Scopus, SciELO, and LILACS. We also searched the register at ClinicalTrials.gov. The search included a combination of subject headings and keywords to represent four main concepts: the Latino population, cancer, family caregiver, and intervention. In addition, we applied a search strategy to eliminate research focused on children. The search strategies for all databases are available in Appendix 1.

### 2.3. Data collection process

The references were exported into Covidence Systematic Review software (https://www.covidence.org; Veritas Health Innovation, Melbourne, Australia) for organization during the review process. Multilingual members of the multidisciplinary research team independently screened the titles and abstracts based on the eligibility criteria; each reference was cross-checked by two coauthors. We repeated the same process during the full-text screening and review phases; each full-text was reviewed by at least two of the coauthors. Conflicts were resolved through weekly team discussions.

### 2.4. Study risk of bias assessment

We used the Cochrane Collaboration's Risk of Bias Tool (Higgins et al., 2011) to assess the quality of RCTs. This tool covers/investigates various domains of bias, such as selection, performance, detection, attrition, and reporting biases. Each domain was assessed with a rating of “low risk,” “high risk,” or “unclear risk” following the guideline's criteria. We used JBI Critical Appraisal Checklist for Quasi-experimental Studies to assess the quality of quasi-experimental studies. This checklist is composed of nine items that can be rated yes, no, unclear or not applicable (Tufanaru et al., 2017). The risk of bias in all included studies was assessed independently by each reviewer before they were cross-checked by at least two reviewers; any conflicts were resolved through ongoing team discussions.

### 2.5. Data extraction and synthesis

The coauthors independently extracted the data relevant to the study aims and cross-checked the data accuracy. Because these studies reported different participant characteristics, intervention components, outcomes, and follow-up assessment timepoints, we were unable to conduct a meta-analysis of their findings. A systematic narrative synthesis has been provided with information presented in the tables to summarize and explain the study, characteristics of participants and interventions, and the outcomes. We compared the extracted results and resolved any discrepancies through ongoing discussions among team members before merging the data.

## 3. Results

### 3.1. Study characteristics

**Figure 1:**
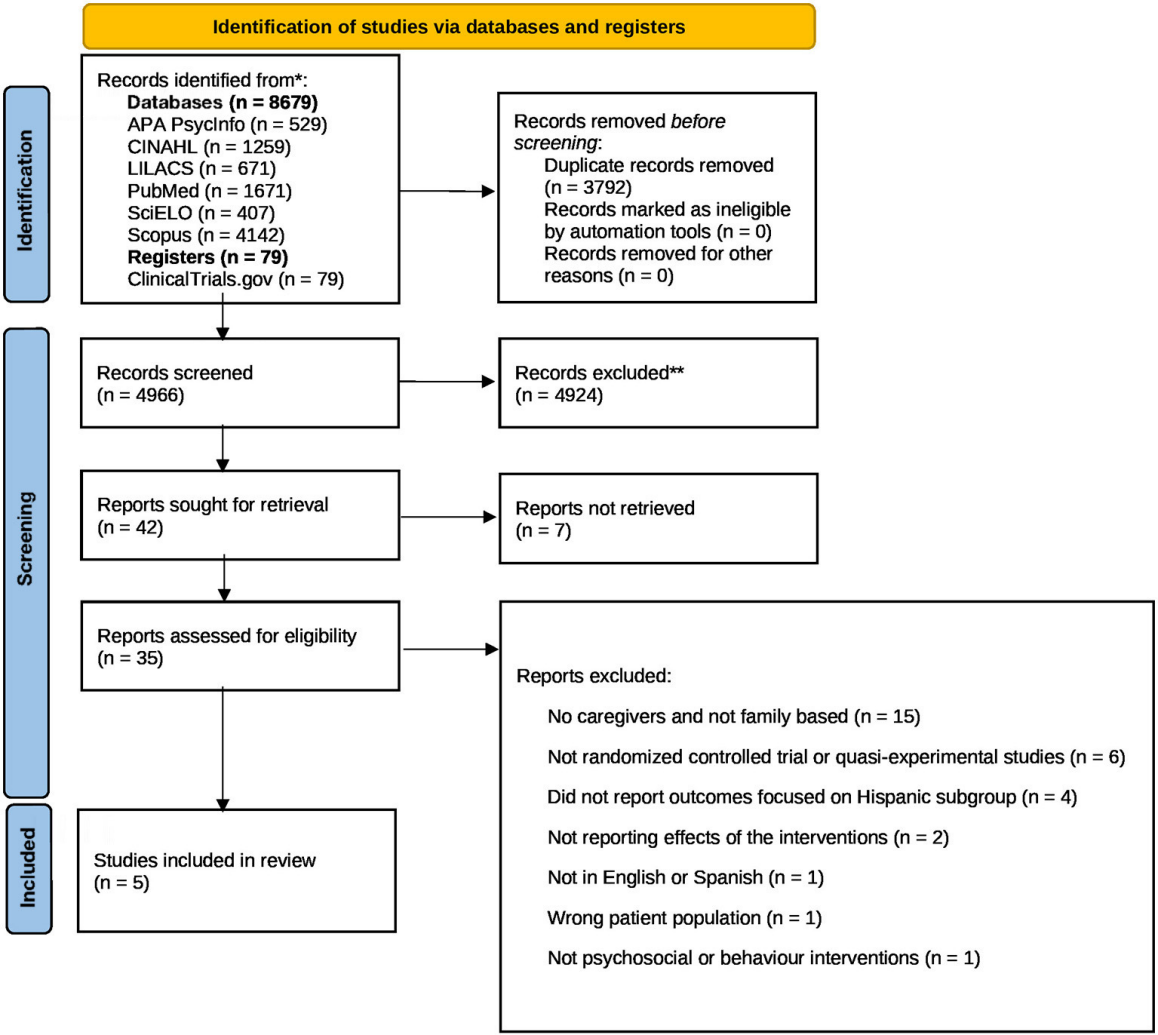
PRISMA 2020 flow diagram for new systematic reviews which included searches of databases and register only. *Consider, if feasible to do so, reporting the number of records identified from each databases or register searched (rather than the total number across all databases/registers). **If automation tools were used, indicate how many records were excluded by a human and how many were excluded by automation tools. From Page et al. (2021). For more information, visit: http://www.prisma-statement.org/.

Of 4,966 studies screened, 35 were considered relevant and were assessed for eligibility. Five studies met the inclusion criteria and were included in the review (Figure 1); four studies were conducted in the U.S., and one was conducted in Brazil. The U.S. study locations were Arizona (Badger et al., 2013, 2020; Crane et al., 2021) and California (Casillas et al., 2021). The Crane study specified that participants also came from the U.S.-Mexican border (Crane et al., 2021). The Brazilian location was São Paulo (Mourao et al., 2017). Three studies were RCTs (Randomized Controlled Trials; Badger et al., 2013, 2020; Crane et al., 2021) and two studies used a quasi-experimental design (i.e., single group pre- and post-test assessments; Mourao et al., 2017; Casillas et al., 2021). Three studies were pilot studies (Badger et al., 2013; Casillas et al., 2021; Crane et al., 2021). All studies focused on both patients and caregivers (dyads), and the sample size ranged from 18 to 230 dyads (Table 1). Regarding participant recruitment, patients were recruited from cancer centers in all the studies (*n* = 5). Additionally, three studies recruited from support groups, and one listed self-referral as a source of sampling (Badger et al., 2013).

**Table 1:** Study and participant characteristics.

**Reference, country**	**Study aim**	**Design^†^**	**Sample size (*N*) Recruitment, retention, and attrition**	**PT age^‡^ Gender Race Cancer type (stage) Household income**	**CG age^§^Gender Race Relationship with PT Household income**
Badger et al. (2013), U.S.	Test the efficacy of two telephone-delivered interventions (interpersonal counseling: TIP-C and health education: THE) in improving QOL (Quality of Life) among patients with breast cancer and their caregivers	RCT	70 PT-CG dyads; 1,233 assessed for eligibility, 90 recruited and randomized, 70 included in analysis	47.34; 100% Female; 100% Hispanic; Breast cancer (Stage I–III) 72.5% <$30,000	42.74; 53.8% Female; 93.8% Hispanic; 45% spouse/significant others, 15% daughters, 14% siblings, 8% friends, 5% mothers, 13% others 66.3% <$30,000
Badger et al. (2020), U.S.	Compare the effectiveness of two 8-week telephone-delivered interventions (TIPC and supported health education—SHE) among patients with breast cancer and their caregivers	RCT	230 PT-CG dyads; 515 assessed for eligibility, 241 consented, 230 randomized and included in analysis	50.71; 100% Female; 100% Hispanic; Breast cancer (Stage II–IV) 73% <$30,000	44.38; Gender: N/A; Race: N/A; 30% spouse/partner, 30% daughters or sons, 15% siblings, 7% mothers, 18% others and friends 57% <$30,000
Casillas et al. (2021), U.S.	Evaluate the potential efficacy of a *photonovela* educational intervention in improving confidence in survivorship care management, decreasing stigma, and increasing knowledge among Latino AYA survivors and their family members	Quasi-experimental	PT = 41, CG = 5; Not reported, but the response rate from enrollment to completion was 100%	20.0 ± 4.1; Gender: N/A; 95% Hispanic, Leukemia/lymphoma, brain/central nervous system, and other solid tumors (N/A) 65.8% <$60,000	43.1 ± 9.6; Gender: N/A; 96.4% Hispanic; 62.5% mothers, 27% fathers, 5.4% brothers; 5.4% spouses/partners 83.9% <$60,000
Crane et al. (2021), U.S.	Test the feasibility, acceptability, and preliminary efficacy of a culturally and linguistically tailored symptom management and lifestyle intervention (SMLI) among Latina cancer survivors and their caregivers	RCT	45 PT-CG dyads; 45 of 72 eligible dyads consented, 16 dyads in the intervention group dropped out, and four dyads in the control group did not complete post-interview	64; 100% Female; 100% Hispanic; Head/neck, liver, breast, colon, kidney, lymphoma, uterine, other cancer, or information missing (N/A) 64.9% <$50,000	53; Gender: N/A; 70% Hispanic; 27% spouses/partners, 11% siblings, 30% children, 5% parents, 27% friends or others 50.0% <$50,000
Mourao et al. (2017), Brazil	Evaluate the effects of Brief Motivational Interview (BMI) on behavioral changes related to social support behavior offered by caregivers to breast cancer patients in chemotherapy	Quasi-experimental	18 PT-CG dyads; Not reported, but 18 dyads were included in data analysis	52.2; 100% Female; Race: N/A; Breast cancer (N/A) N/A	N/A except aged over 18 years

† RCT, Randomized Controlled Trial.

‡ PT, Patients with cancer.

§ CG, Adult caregivers, including family members (Casillas) and/or friends (Crane), or any individuals designated as such (Mourao). CG was termed as “supportive partner” in Badger et al. (2013) and “anyone in the social network” in Badger et al. (2020), N/A, Not available.

### 3.2. Risk of bias assessment

Evaluated based on the Cochrane Collaboration's Risk of Bias Tool, two studies were found to have a low risk of bias. The other one study was deemed to have an unclear risk of bias because they did not describe allocation concealment, blinding of participants or personnel, and blinding of outcome assessment. The two quasi-experimental studies based on the JBI Critical Appraisal Checklist for Quasi-experimental Studies reached at five of nine points on the JBI checklists (Table 2).

**Table 2:** Assessment of study quality based on published data using Cochrane collaboration's criteria and JBI critical appraisal checklist for quasi-experimental studies.

**Assessment of study quality using Cochrane collaboration's criteria for RCTs**									
**References**	**Random sequence generation**	**Allocation concealment**	**Blinding of participants and personnel**	**Blinding of outcome assessment**	**Incomplete outcome data**	**Selective reporting**	**Level of risk**		
Badger et al. (2013)	U	U	U	U	L	L	U		
Badger et al. (2020)	L	L	L	L	L	L	L		
Crane et al. (2021)	L	U	U	L	L	L	L		
**Assessment of study quality using JBI critical appraisal checklist for quasi-experimental studies**									
	**Clear in the cause and effect**	**Participants Included in comparisons similar**	**Participants included in comparisons receiving similar treatment/care**	**There was a control group**	**Multiple measurements of the outcome**	**Follow up was complete**	**Outcomes of participants included in comparisons measured in the same way**	**Outcomes measured in a reliable way**	**Statistical analysis used was appropriate**
Casillas et al. (2021)	Y	N/A	N/A	N	Y	Y	N/A	Y	Y
Mourao et al. (2017)	Y	N/A	N/A	N	Y	Y	N/A	Y	Y

U, Unclear; L, Low risk; N/A, Non-applicable; Y, Yes; N, No.

### 3.3. Participant characteristics

Three studies included patients diagnosed with breast cancer (Badger et al., 2013, 2020; Mourao et al., 2017), and two included patients with mixed types of cancer at (Casillas et al., 2021; Crane et al., 2021). The mean ages across all studies ranged between 20 and 64 years old for patients and between 43 and 53 years old for the caregivers. Four studies included female patients only (Badger et al., 2013, 2020; Mourao et al., 2017; Crane et al., 2021). The caregivers were spouses/partners, children, siblings, parents, and friends (Table 1). The studies conducted in the U.S. (Badger et al., 2013, 2020; Casillas et al., 2021; Crane et al., 2021) reported that at least 94% of the participants self-identified as Latinos. Among four studies reported household income, most patients and caregivers had a household income <$60, 000. Only one study reported patients' access to health care and found that most patients regularly visited a survivorship clinic for care (Casillas et al., 2021).

### 3.4. Intervention characteristics

Table 3 summarizes the interventions' characteristics. The interventions primarily aimed to improve quality of life for patients and caregivers (Badger et al., 2013, 2020), decrease anxiety and depression (Badger et al., 2020), and support survivorship and behavioral changes in patients and caregivers (Mourao et al., 2017; Casillas et al., 2021; Crane et al., 2021). The conceptual frameworks or theories used to support the interventions included the Stress Process Model (Badger et al., 2020), the Social Cognitive Theory (Crane et al., 2021), and the Brief Motivational Interviewing (Mourao et al., 2017).

**Table 3:** Intervention characteristics.

**References**	**Intervention**				
	**Theoretical basis**	**Intervention component**	**Cultural consideration**	**Delivery mode; format; duration; dosage** ^†^	**Interventionist**
Badger et al. (2013)	Standard interpersonal psychotherapy	Telephone interpersonal counseling (TIP-C) addressed (1) mood and affect management, (2) emotional expression, (3) interpersonal communication and relationships, (4) social support, and (5) cancer information. Telephone health education group (THE) focused on (1) breast health and breast cancer, tests for diagnosis, and prevention and associated terminology; (2) treatment, related side effects, and strategies to manage the side effects; (3) lifestyle interventions (e.g., nutrition and physical activity); and (4) referrals and resources.	Interventions tailored to the cultural values and beliefs of participants about the importance of family and close friends (*la familia*) in health outcomes. Integrated the core cultural values of *respeto* (mutual respect), *confianza* (relationship of trust), *personalismo* (valuing personal relationships and personal familiarity), and *espiritu* (spirit) into contacts with the participants. Involved bilingual, bicultural research staff to decrease distrust (an aspect of *confianza*) of culturally alien institutions.	Telephone and printed materials Family 8 weeks N/A	TIP-C: bilingual, bicultural master's prepared social worker THE: bilingual bicultural paraprofessionals
Badger et al. (2020)	Standard interpersonal psychotherapy	TIPC addressed (1) mood and affect management, (2) emotional expression, (3) interpersonal communication and relationships, (4) social support, and (5) cancer information, resources, and referral. Supported health education (SHE) focused on (1) breast health and breast cancer, (2) routine tests and associated terminology, (3) treatment, side effects, and strategies to manage side effects, (4) healthy lifestyle (e.g., nutrition and physical activity), and (5) resources and referrals.	Interventions tailored to the cultural values and beliefs, particularly with respect to the importance of family and close friends (*familism*). Incorporated in contacts with participants to reflect the core values of *respeto* (displaying mutual respect), *personalismo* (valuing personal relationships and personal familiarity by having the same interventionist each time), *simpatia* (being pleasant and polite on the phone and in all interactions), *confianza* (a relationship of trust). There is a distrust of alien institutions (decreased by presence in the Latino/a cancer community and use of bilingual bicultural research staff).	Telephone and printed materials Individual 8 weeks ~30 min each session	Trained research personnel
Casillas et al. (2021)	N/A	Each *photonovela* intervention session was led by a health advocate who assigned a character role to each participant. Upon completion, the survivors or family members could ask questions regarding the content, and care planning goals were determined by the survivor through discussion with their family.	Intervention targeted the specific care needs that are culturally relevant to Latino adolescent and young adult cancer survivors (AYA) and educated AYA on their need for survivorship care through engaging the whole nuclear family.	Face-to face and booster phone call Family 6 weeks 1 meeting + 1 booster call	Trained health advocates
Crane et al. (2021)	Social Cognitive Theory	Motivational interviewing (MI) served as the basis for the behavior-change strategy in the weekly coaching sessions. Coaches and participants set up Specific, Measurable, Attainable, Relevant, and Timely (SMART) goals. Printed materials from the Symptom Management and Survivorship Handbook (SMSH) Fitbit^®^ for self-monitoring (e.g., daily steps or active minutes) and coach used the information to tailor the intervention sessions.	Intervention was culturally and linguistically tailored to Latina cancer survivors and their informal caregivers. Interventionists were bicultural health coaches. Measurements/questionnaires were translated into Spanish.	Telephone interviews, text messaging, and Fitbit Individual 12 weeks 20–30 min coaching sessions	Bicultural health coaches
Mourao et al. (2017)	Brief Motivational Interviewing (BMI)	Applying BMI techniques through simulations of situations involving patients, the intervention was applied to the caregivers only. Weekly meetings 1–3 defined which support behaviors caregivers should adopt; caregivers gave feedback about the intervention received at the 4th meeting. BMI had six elements: (1) return, (2) patient's personal responsibility, (3) clear advice for changing habit, (4) selection of a specific treatment approach but offering alternative strategies, (5) therapist's empathy, and (6) strengthening the self-efficacy of patient's hope.	Intervention was developed by Brazilian personnel. The Brazilian instruments were factor analyzed.	In-person meetings with individual caregiver 4 weeks Each session lasting 30 min	Trained study personnel

† N/A, Not available.

A common element for all the U.S. publications was to include cultural considerations in their interventions. These considerations were mostly to incorporate Latino cultural values and beliefs in the intervention, such as the importance of the family in health outcomes (Badger et al., 2013, 2020; Casillas et al., 2021; Crane et al., 2021), as well as personalismo, which is understood as valuing personal familiarity. Personalismo was honored by having the same interventionist each time (Badger et al., 2013, 2020). For these four studies, the interventions and questionnaires were translated into Spanish. Cultural relevance/adaptation also included the use of bilingual, bicultural interventionists who were master's-prepared social workers and paraprofessionals (Badger et al., 2013), health coaches (Crane et al., 2021), health advocates (Casillas et al., 2021), and other trained personnel (Badger et al., 2020). Mourao's study did not report cultural considerations, but the intervention was made from Brazilian study personnel to Brazilian participants–thus, keeping their own cultural elements and the Portuguese language (Mourao et al., 2017).

All studies included interventions that provided education and support directed at caregivers or patients with caregivers, including knowledge and resources related to illness, treatment and care, wellness and lifestyle recommendations, and stress and symptom management. Two interventions also included socio-emotional support (Badger et al., 2013, 2020). The intervention components, techniques, and delivery modes included telephone interpersonal counseling (Badger et al., 2013, 2020); in-person photonovelas and a booster phone call (Casillas et al., 2021); weekly coaching to dyads plus written materials and a Fitbit for self-monitoring (Crane et al., 2021); and in-person private meetings for education of brief motivational interviews (Mourao et al., 2017). Only one studies' durations ranged from 4 weeks (Mourao et al., 2017) to 7 months (Casillas et al., 2021), whereas the intervention frequency ranged from once (Casillas et al., 2021) to 12 weekly sessions (Crane et al., 2021). Four interventions provided the same components for patients and caregivers but differed by delivery format and doses. For example, the photonovalas intervention was delivered for patients and caregivers at the same time (Casillas et al., 2021). The socio-emotional support intervention was provided separately to patients and caregivers to allow them to have their own discussion space (Badger et al., 2020). Another socio-emotional support intervention provided more doses for patients than caregivers (Badger et al., 2013). The lifestyle intervention could be delivered either separately for patients and caregivers or both (Crane et al., 2021). The intervention using the brief motivational interviews was delivered to caregivers only (Mourao et al., 2017).

### 3.5. Intervention outcomes

Table 4 summarizes the intervention measurements and study outcomes.

**Table 4:** Measurements and study outcomes.

**References**	**Target outcomes^†^**	**Measurements^‡^**	**Assessment time points^§^**	**Results^¶^**
Badger et al. (2013)	QOL outcomes: 1) Psychological distress (depression, negative affect, stress, and anxiety) 2) Physical wellbeing (fatigue and symptom distress) 3) Social wellbeing 4) Spiritual wellbeing	Symptoms of depression: Center for Epidemiological Studies-Depression Scale; Negative affect: negative affect subscale of the Positive and Negative Affect Schedule; Stress: Perceived Stress Scale; Anxiety: State-Trait Anxiety Inventory; Fatigue: Multidimensional Fatigue Inventory; Distress: General Symptom Distress Scale; Social wellbeing: social wellbeing subscale of the QOL—Breast Cancer instrument; Spiritual wellbeing: spiritual wellbeing subscale of the QOL—Breast Cancer instrument.	BL, 8 weeks after intervention, and 16 weeks after intervention	Both patients and caregivers had significant improvements in all dimensions of QOL over the 16 weeks of the investigation in both intervention groups (TIP-C and THE). No evidence of the superiority of either intervention for improving QOL.
Badger et al. (2020)	QOL outcomes: 1) Psychological distress (depression, negative affect, stress, and anxiety) 2) Symptoms, symptom distress, and symptom management 3) Social support and social isolation	Depression and anxiety: PROMIS short forms; Symptoms: General Symptom Distress Scale; Social support and social isolation: PROMIS short forms, including informational support, emotional support, and social isolation.	BL, 2, 4, and 6 months after intervention	TIPC was superior to SHE for the management of depression. Survivors had significantly lower depression in TIPC compared to SHE immediately post-intervention. SHE was more successful than TIPC in the management of anxiety, social isolation, and cancer-related symptoms.
Casillas et al. (2021)	1) Confidence in survivorship care management 2) Cancer stigma 3) Knowledge related to late effects and the need for consistent cancer survivorship care	Confidence in survivorship care management: authors constructed based on the Health Belief Model; Cancer stigma: authors adapted based on existing ones for other diseases, such as irritable bowel syndrome; Knowledge related to late effects: 10 true/false items related to late effects and cancer survivorship care.	BL, post-intervention, and during the booster call (6 weeks to 7 months after intervention)	For cancer survivors at both the follow-up and booster: confidence in survivorship care significantly increased; cancer stigma did not significantly decrease; knowledge did not significantly increase. For family members: confidence significantly increased at the follow-up assessment (*p* < 0.05) but not at the booster; cancer stigma did not significantly decrease at the follow-up, but significantly increased at the booster (*p* < 0.05); knowledge did not significantly increase at the follow-up, but significantly increased at the booster (*p* < 0.05).
Crane et al. (2021)	1) Feasibility and acceptability 2) Preliminary efficacy: Primary outcomes: diet and physical activity. Secondary outcomes: severity of symptoms and self-efficacy for symptom management	Feasibility: consent rate Acceptability: percentage of intervention dyads completing at least 75% of sessions and qualitative feedback (open-ended questions) Diet: USDA Food Security Questionnaire and NCI Dietary Screener Questionnaire Physical activity: Women's Health Initiative Physical Activity Questionnaire Severity of symptoms: General Symptom Distress Scale Self-efficacy: PROMIS Self-efficacy questionnaire.	BL, weekly assessments for 12 and 13 weeks after BL	Feasibility: 63% consent rate. Acceptability: 86% of intervention dyads completed at least 75% of sessions; Both survivors and caregivers expressed satisfaction with the intervention and praised the health coaches. Preliminary efficacy: Intervention arm had improved mean scores for goal attainment of lifestyle behaviors for survivors and caregivers. Diet and Physical activity: Survivors: medium-to-large effect sizes for servings of total fruits, vegetables, and sugar intake. Medium clinically significant effect sizes for total minutes of physical activity per week and grams of fiber intake per day. Caregivers: medium to large-intervention effects for total sugar intake and sugar intake from sugar-sweetened beverages, and a medium intervention effect for vegetable intake. Severity of Symptoms: Survivors: Medium-to-large intervention effects for improved summed symptom severity. Small effects for global symptom distress and self-efficacy for managing symptoms Caregivers: no intervention effect for any of the symptom measures.
Mourao et al. (2017)	Perceived social support	Perception of structural and emotional social support: Brazilian Social Support Scale (SSS) questionnaire. The Record and Self-monitoring form (FRAM) was used by caregivers to track progress of the BMI concepts and collected in 7-day intervals. FARM was also used to define which support behaviors should be adopted by the caregivers or not	SSS: BL and 1 month after BL FRAM: 7-day intervals	All 10 items of the SSS were statistically significant except for Question 1, showing a positive impact of the intervention. Higher social support, both in the emotional and instrumental dimensions, among women with breast cancer undergoing chemotherapy after the intervention

† QOL, Quality of Life.

‡ PROMIS, Patient Reported Outcome Measurement Information System.

§ BL, Baseline.

¶ TIP-C, Telephone interpersonal counseling; THE, Telephone health education; SHE, Supported health education.

#### 3.5.1. Improving quality of life

Two studies conducted by Badger's team compared the effectiveness of two psychosocial interventions for improving quality-of-life outcomes in Latinas with breast cancer and their caregivers (Badger et al., 2013, 2020). Supportive health education intervention was more successful than the telephone interpersonal counseling intervention in improving quality-of-life outcomes, including social isolation and cancer-related symptoms, among breast cancer survivors (Badger et al., 2020). However, when telephone interpersonal counseling was compared with telephone health education intervention, greater improvement in all quality-of-life dimensions in both intervention groups was reported for patients and caregivers over time, and no evidence documented the superiority of either intervention (Badger et al., 2013).

#### 3.5.2. Decreasing anxiety and depression

Badger's study found that telephone interpersonal counseling was superior to supportive health education in reducing depression in breast cancer survivors and caregivers. Yet supported health education was more successful in managing anxiety than telephone interpersonal counseling in caregivers (Badger et al., 2020).

#### 3.5.3. Supporting survivorship and behavioral changes

In their study evaluating the effectiveness of motivational interviewing offered to the caregivers, Mourao et al. found that patients with breast cancer reported higher levels of perceived social support (e.g., emotional and instrumental support) after the intervention (Mourao et al., 2017). Casillas' study assessed a “photonovela” educational intervention to increase knowledge and engage patients and caregivers in survivorship care (Casillas et al., 2021). The intervention was effective in increasing knowledge about and confidence in managing late effects of cancer and issues related to survivorship care among Latino adolescent and young adult cancer survivors and their family members (Casillas et al., 2021). Crane's study evaluated the feasibility, acceptability, and preliminary efficacy of an integrated symptom management and lifestyle intervention to improve adherence to the American Cancer Society's Guidelines on Nutrition and Physical Activity in Latina cancer survivors and their informal caregivers. The researchers reported that 63% of approached dyads consented to attend the intervention, and the intervention was acceptable for 98% of dyads. The preliminary efficacy results indicated that lifestyle behaviors improved for participants in the intervention group as compared to the control group: specifically, medium-to-large effects for some dietary changes among survivors and caregivers and a reduction of symptom burden among survivors (Crane et al., 2021).

## 4. Discussion

To our knowledge, this is the first systematic review of studies that evaluates family-based psychosocial interventions for adult Latino patients with cancer and their caregivers. These interventions were conducted in western U.S. states and Brazil. Cultural sensitivity has been considered in the intervention design and implementation, including the diverse range of family members as caregivers; cultural adaptations of intervention content and methods; and bilingual interventionists. Most interventions have used printed materials plus in-person or telephone education, support, and counseling; one pilot study also integrated Fitbit for monitoring. These interventions have shown consistent effects in improving quality of life and enhancing perceived survivorship support for patients and caregivers, but varied effects in reducing anxiety and depression. There is also promising evidence to support the feasibility and acceptability of conducting eHealth interventions to facilitate healthy lifestyle changes. However, there is a need for rigorous research on the growing population of Latino patients with cancer and their caregivers.

Our systematic review noticed the culturally sensitive considerations in the intervention design and implementation (e.g., intervention content, modes of delivery, and interventionist) to best meet the needs of Latino patients and their caregivers. These cultural adaptations included standardized educational materials in Spanish (Badger et al., 2013); using face-to-face and telephone methods to deliver the interventions; and involving culturally competent bilingual interventionists (Badger et al., 2013; Crane et al., 2021), all of which are crucial for removing the barriers to accessing supportive care and for facilitating intervention delivery to Latino population. Especially the intervention conducted in Brazil arranged the meeting in the place of caregiver preference (e.g., residence of caregivers) if they were not at the oncology center, which made it easily accessible. We also found that Latino sociocultural values have compelled a diverse range of family members into the caregiver role: spouses/partners, children, parents, siblings, friends, and others. This finding is different from those of a systematic review of psychosocial, behavioral interventions for patients with cancer and their family caregivers (75.9% of the patients and caregivers were Caucasians) that two-thirds of the studies included spouses and partners as caregivers (Song et al., 2021). This element is supported by the familism as one of the most important values of Latinos (Badger et al., 2020). As expected, family-based psychosocial interventions had beneficial effects on multiple aspects of the psychosocial outcomes for both Latino patients and their caregivers. Specifically, these interventions had consistent positive effects on quality of life in terms of social isolation, cancer-related symptoms, and emotional and social/family wellbeing; reduced anxiety and depression; changed health behaviors; increased cancer knowledge; and enhanced self-efficacy and social support. Consistent with previous evidence, these findings confirm that psychosocial interventions positively influence the psychosocial outcomes of patients and family caregivers (Northouse et al., 2010; Gabriel et al., 2020). Also, Badger's team further evaluated the cost and efficacy of two psychosocial interventions and found reductions in urgent care and emergency department visits among Latino patients in a supportive health education group as compared with those receiving telephone interpersonal counseling (Badger et al., 2021). However, these findings should be interpreted with caution because three of the five studies were still at the pilot study stage with small sample sizes or without control groups. More high-quality studies are necessary to validate these findings.

Although the interventions were culturally attuned and showed positive effects, this review also identified methodological limitations and risks of bias in these studies. For example, few studies described allocation concealment, whether blinding of participants or research personnel, and there was little information on the blinding of outcome assessment. Additionally, the small sample sizes of most of the studies have made it difficult to draw definitive conclusions about the true intervention effects. The quality of the family-based intervention studies could be improved by standardizing the research methods and reporting as recommended by the Cochrane Collaboration's Risk of Bias Tool (Higgins et al., 2011) and using sufficiently powered, more geographically diverse Latino samples.

It is also concerning to find that the current family-based intervention research has narrow foci. For example, four out of the five interventions identified in this review focused on middle-aged female Latino patients with cancer (mostly breast cancer) and their caregivers. All studies reported the intervention effects on health outcomes of individual patients and caregivers rather than outcomes at dyadic level or at the healthcare system levels. Furthermore, the four interventions published in English were conducted in California and Arizona, a region where Latino communities are heavily Hispanic and Latino Americans (U.S. department of Health and Human Services Office of Minority Health, 2021); only one study was from Latin America, conducted in Brazil. Different subgroups of Latino populations are disproportionally burdened by cancer (Miller et al., 2021), especially infection-related cancers. Hispanics have higher incidence rates for cancers of the cervix, stomach, liver, and gall bladder than non-Hispanic Whites; Hispanic men (but not Hispanic women) have a significantly elevated risk of gastric cancer (Haile et al., 2012). While Latinos of different country origins have varied cancer incidence and mortality rates (Pinheiro et al., 2017), and Latinos of different genders and sociodemographic backgrounds (e.g., age, education, and insurance) also engage in health behaviors differently and have varied access to care, all of which can adversely affect cancer incidence and mortality (American Cancer Society, 2008). The negative impact of social determinants of health can further negatively affect the health outcomes of Latino patients with cancer and their caregivers (American Cancer Society, 2021). In addition, Latino cancer patients reported higher physical and psychological symptoms such pain, fatigue, distress than non-Latino white counterparts, which significant influence their quality of life (Eversley et al., 2005; Alcalá, 2014). All of these disparities point to the urgent need for research and supportive psychosocial care interventions, for the growing population of Latino patients of both genders and with diverse types of cancer and their caregivers, who manage cancer treatment, and their effects in diverse contexts of the social determinants of health during their survivorship.

Even though our systematic review only included five family-based psychosocial interventions focused on Latino populations, we found a steady growth in studies focused on psychosocial needs among Latino patients with cancer and/or their family caregivers during the past decade. In the full-text screening phase, we also noticed that some research teams were developing the interventions or collecting data. For example, one team in M.D. Anderson Cancer Center planned to evaluate the feasibility of using a positive-activities intervention to improve the psychological and interpersonal wellbeing of cancer patients and their caregivers from collectivist cultures (e.g., Latinos, Asian Americans, and African Americans; U.S. National Library of Medicine, 2021a). Another team at the University of Arizona proposed an RCT to test the effects of a symptom management and lifestyle intervention on improving the vegetable and fruit intakes among Hispanic female cancer survivors and their caregivers (U.S. National Library of Medicine, 2021b).

This systematic review has some limitations. First, all the studies were conducted in North America (USA) and South America (Brazil). Therefore, the results cannot be generalized to the other parts of the world, including the other countries of South America, where the language and the culture are different. Second, due to the small sample size and methodological heterogeneity of the included studies, we cannot conduct a quantitative synthesis of the intervention effects. As there are many studies focused on non-Hispanic populations, the findings of this review should be considered in the larger context of family-based intervention research. In addition, this systematic review focused on family-based interventions. We, thus, excluded interventions that focused on either patients or caregivers but didn't report the outcomes of both patients and caregivers, which might be beneficial for patients or caregivers as suggested by other published review (McNulty et al., 2016). Lastly, we conducted a systematic search of peer-reviewed publications in the six databases. Relevant studies that were not published in the peer-reviewed journals might have been missed.

Nevertheless, this systematic review also has several strengths. We conducted a rigorous literature search in the six major databases from their dates of inception through June 2022 and included the literature published in English, Spanish, and Portuguese. Based on a comprehensive review and synthesis of the existing family-based psychosocial interventions, this review provides valuable information that will help advance this line of research and ultimately enhance supportive oncologic care for Latino cancer patients and their family caregivers.

### 4.1. Recommendations

To facilitate the development of future family-based psychosocial intervention research for Latino adult patients with cancer and their family caregivers, we make the following recommendations:

- Acknowledging a family's role when researchers begin to design a supportive oncologic care intervention.
- Incorporating culturally and linguistically competent interventionists to ensure that participants are comfortable and able to engage in the programs.
- Providing linguistically appropriate interventions (all study-related materials and communications) to remove language barriers.
- Conducting rigorously designed research that uses common data elements to help synthesize future evidence of intervention effects.
- Developing interventions that target non-gender specific types of cancer and recruit both male and female Latino patients and their caregivers.
- Recruiting socioeconomically and geographically diverse populations of patients and caregivers who have diverse social determinants of health to further test the interventions, including those presented in this review.
- Incorporating intervention components and evaluation methods for non-Hispanic groups into future family-based research among Hispanic population.

## 5. Conclusion

This systematic review identified the characteristics and effects of family-based psychosocial interventions for adult Latino patients with cancer and their caregivers. Findings from this systematic review help to deepen our understanding of the family-based interventions for the Latino population managing cancer. Future culturally appropriate family-based interventions are needed to design and evaluate to help Latino families with diverse backgrounds cope with cancer.

## Data availability statement

The original contributions presented in the study are included in the article/Supplementary material, further inquiries can be directed to the corresponding author.

## Author contributions

LS: study conception. LS, TG, JC, and SX: study design. LS, TG, PC, LR, JC, AA, and YP: first draft of the manuscript. All authors: data collection, synthesis, reviewed the drafts, provided feedback and comments to improve the manuscript quality, and read and approved the final manuscript.

## References

[B1] Alcalá H. E. (2014). Differential mental health impact of cancer across racial/ethnic groups: Findings from a population-based study in California. BMC Public Health. 14, 1–9. 10.1186/1471-2458-14-930 25200245 PMC4175189

[B2] American Cancer Society (2008). South Atlantic Cancer Facts and Figures 2008. Available online at: https://dph.georgia.gov/document/document/acs-south-atlantic-division-cancer-facts-figures-2008/download (accessed July 9, 2022).

[B3] American Cancer Society (2021). Cancer Facts and Figures for Hispanic/Latino People 2021–2023. Available online at: https://www.cancer.org/content/dam/cancer-org/research/cancer-facts-and-statistics/cancer-facts-and-figures-for-hispanics-and-latinos/hispanic-latino-2021-2023-cancer-facts-and-figures.pdf (accessed July 9, 2022).

[B4] Badger T. A. Segrin C. Hepworth J. T. Pasvogel A. Weihs K. Lopez A. M. (2013). Telephone-delivered health education and interpersonal counseling improve quality of life for Latinas with breast cancer and their supportive partners. Psychooncology 22, 1035–1042. 10.1002/pon.3101 22573418

[B5] Badger T. A. Segrin C. Sikorskii A. Pasvogel A. Weihs K. Lopez A. M. . (2020). Randomized controlled trial of supportive care interventions to manage psychological distress and symptoms in Latinas with breast cancer and their informal caregivers. Psychol. Health 35, 87–106. 10.1080/08870446.2019.1626395 31189338

[B6] Badger T. A. Sikorskii A. Segrin C. Given C. W. (2021). Supportive health education reduces health care utilization and costs in Latinas with breast cancer and their caregivers. Support Care Cancer 29, 1225–1233. 10.1007/s00520-020-05593-9 32613374

[B7] Casillas J. N. Schwartz L. F. Gildner J. L. Crespi C. M. Ganz P. A. Kahn K. L. . (2021). Engaging Latino adolescent and young adult (AYA) cancer survivors in their care: Piloting a photonovela intervention. J. Cancer Educ. 36, 971–980. 10.1007/s13187-020-01724-2 32333369 PMC10132777

[B8] Costas-Muñiz R. Hunter-Hernández M. Garduño-Ortega O. Morales-Cruz J. Gany F. (2017). Ethnic differences in psychosocial service use among non-Latina white and Latina breast cancer survivors. J. Psychosoc. Oncol. 35, 424–437. 10.1080/07347332.2017.1310167 28332946 PMC5647778

[B9] Crane T. E. Badger T. A. O'Connor P. Segrin C. Alvarez A. Freylersythe S. J. . (2021). Lifestyle intervention for Latina cancer survivors and caregivers: the Nuestra Salud randomized pilot trial. J. Cancer Surviv. 15, 607–619. 10.1007/s11764-020-00954-z 33170481 PMC12257549

[B10] Creamer J. (2020). Inequalities Persist Despite Decline in Poverty For All Major Race and Hispanic Origin Groups. Available online at: https://www.census.gov/library/stories/2020/09/poverty-rates-for-blacks-and-hispanics-reached-historic-lows-in-2019.html (accessed July 9, 2022).

[B11] Eversley R. Estrin D. Dibble S. Wardlaw L. (2005). Post-treatment symptoms among ethnic minority breast cancer survivors. Oncol. Nurs. Forum. 32, 250–256. 10.1188/05.ONF.250-256 15759063

[B12] Gabriel I. Creedy D. Coyne E. (2020). A systematic review of psychosocial interventions to improve quality of life of people with cancer and their family caregivers. Nurs. Open 7, 1299–1312. 10.1002/nop2.543 32802350 PMC7424465

[B13] Haile R. W. John E. M. Levine A. J. Cortessis V. K. Unger J. B. Gonzales M. . (2012). A review of cancer in U.S. Hispanic populations. Cancer Prev. Res. 5, 150–163. 10.1158/1940-6207.CAPR-11-0447 22307564 PMC5815320

[B14] Higgins J. P. Altman D. G. Gotzsche P. C. Juni P. Moher D. Oxman A. D. . (2011). The Cochrane Collaboration's tool for assessing risk of bias in randomised trials. Br. Med. J. 343, d5928. 10.1136/bmj.d5928 22008217 PMC3196245

[B15] King J. J. Segrin C. Badger T. A. Thomson C. A. (2022). Exploring the relationship between loneliness, spirituality, and health-related quality of life in Hispanic cancer caregivers. Support Care Cancer 22, 5. 10.1007/s00520-022-06800-5 35142912 PMC9046141

[B16] McNulty J. Kim W. Thurston T. Kim J. Larkey L. (2016). Interventions to improve quality of life, well-being, and care in Latino cancer survivors: A systematic literature review. Oncol. Nurs. For. 43, 374–384. 10.1188/16.ONF.374-384 27105198

[B17] Miller K. D. Ortiz A. P. Pinheiro P. S. Bandi P. Minihan A. Fuchs H. E. . (2021). Cancer statistics for the US Hispanic/Latino population, 2021. CA Cancer J. Clin. 71, 466–487. 10.3322/caac.21695 34545941

[B18] Moadel A. B. Morgan C. Dutcher J. (2007). Psychosocial needs assessment among an underserved, ethnically diverse cancer patient population. Cancer 109, 446–454. 10.1002/cncr.22357 17123273

[B19] Mourao C. M. L. Fernandes A. F. C. Moreira D. P. Martins M. C. (2017). Motivational interviewing in the social support of caregivers of patients with breast cancer in chemotherapy. Rev. Esc. Enferm. USP 51, e03268. 10.1590/s1980-220x2017001803268 29267741

[B20] Nahleh Z. A. (2016). Decreased health related quality of life among Hispanic breast cancer survivors. Women's Health 2, 16. 10.15406/mojwh.2016.02.0001618956514

[B21] National Alliance for Caregiving (2019). Hispanic Family Caregiving in the U.S. Available online at: https://www.caregiving.org/wp-content/uploads/2020/05/Hispanic_Caregiver_Study_web_ENG_FINAL_11_04_08.pdf (accessed July 9, 2022).

[B22] Northouse L. L. Katapodi M. C. Song L. Zhang L. Mood D. W. (2010). Interventions with family caregivers of cancer patients: Meta-analysis of randomized trials. CA Cancer J. Clin. 60, 317–339. 10.3322/caac.20081 20709946 PMC2946584

[B23] Page M. J. McKenzie J. E. Bossuyt P. M. Boutron I. Hoffmann T. C. Mulrow C. D. . (2021). The PRISMA 2020 statement: An updated guideline for reporting systematic reviews. Br. Med. J. 372, n71. 10.1136/bmj.n71 33782057 PMC8005924

[B24] Pinheiro P. S. Callahan K. E. Gomez S. L. Marcos-Gragera R. Cobb T. R. Roca-Barcelo A. . (2017). High cancer mortality for US-born Latinos: Evidence from California and Texas. BMC Cancer 17, 478. 10.1186/s12885-017-3469-0 28693448 PMC5504850

[B25] Shen M. J. Gonzalez C. Leach B. Maciejewski P. K. Kozlov E. Prigerson H. G. (2020). An examination of Latino advanced cancer patients' and their informal caregivers' preferences for communication about advance care planning: A qualitative study. Palliat. Support Care 18, 277–284. 10.1017/S1478951519000890 31699175 PMC7205556

[B26] Siefert M. L. Williams A. L. D. M. F. Chappel-Aiken L. McCorkle R. (2008). The caregiving experience in a racially diverse sample of cancer family caregivers. Cancer Nurs. 31, 399–407. 10.1097/01.NCC.0000305760.04357.96 18772665 PMC2771410

[B27] Skiba M. B. Lopez-Pentecost M. Werts S. J. Ingram M. Vogel R. M. Enriquez T. . (2022). Health promotion among Mexican-origin survivors of breast cancer and caregivers Living in the United States–Mexico border region: Qualitative analysis from the Vida Plena Study. JMIR Cancer 8, e33083. 10.2196/33083 35200150 PMC8914737

[B28] Song L. Qan'ir Y. Guan T. Guo P. Xu S. Jung A. . (2021). The challenges of enrollment and retention: A systematic review of psychosocial behavioral interventions for patients with cancer and their family caregivers. J. Pain Symptom. Manag. 4, 19. 10.1016/j.jpainsymman.2021.04.019 33933618 PMC8419067

[B29] Tufanaru C. Munn Z. Aromataris E. Campbell J. Hopp L. (2017). “Chapter 3: Systematic reviews of effectiveness,” in Joanna Briggs Institute Reviewer's Manual, eds E. Aromataris, Z. Munn (The Joanna Briggs Institute). Available online at: https://reviewersmanual.joannabriggs.org/ (accessed July 9, 2022).

[B30] U.S. department of Health Human Services Office of Minority Health (2021). Profile: Hispanic/Latino Americans. Available online at: https://minorityhealth.hhs.gov/omh/browse.aspx?lvl=3&lvlid=64 (accessed July 9, 2022).

[B31] U.S. National Library of Medicine (2021a). Positive Activities Intervention to Improve Quality of Life in Collectivist Culture Cancer Patients and Their Caregivers. Available online at: https://beta.clinicaltrials.gov/study/NCT04810052?patient=positive%20activities%20intervention&locStr=&distance=0 (accessed July 9, 2022).

[B32] U.S. National Library of Medicine (2021b). SMLI With Hispanic Cancer Survivors and Caregivers. Available online at: https://clinicaltrials.gov/ct2/show/NCT05364372 (accessed July 9, 2022).

[B33] Valdivieso-Mora E. Peet C. L. Garnier-Villarreal M. Salazar-Villanea M. Johnson D. K. (2016). A systematic review of the relationship between familism and mental health outcomes in Latino population. Front. Psychol. 7, 1632. 10.3389/fpsyg.2016.01632 27826269 PMC5078495

[B34] Wells J. N. Cagle C. S. Bradley P. Barnes D. M. (2008). Voices of Mexican American caregivers for family members with cancer: On becoming stronger. J. Transcult. Nurs. 19, 223–233. 10.1177/1043659608317096 18403715

