# Supplementary material for: Family-based psychosocial interventions for adult Latino patients with cancer and their caregivers: A systematic review
Source: Front Psychol. 2023 Mar 30;14:1052229. doi: 10.3389/fpsyg.2023.1052229 (PMC10097880; doi:10.3389/fpsyg.2023.1052229)
Supplement: Supplementary file 1 [file Data_Sheet_1.docx]

**Appendix 1. Searching strategy for all databases**

## Database: PubMed (MEDLINE)

| Set # |  | Results |
| --- | --- | --- |
| 1 | “Hispanic Americans”[MeSH Terms] OR "Central America"[Mesh] OR "Cuba"[Mesh] OR "Dominican Republic"[Mesh] OR "Latin America"[Mesh] OR "Mexico"[Mesh] OR "Puerto Rico"[Mesh] OR "South America"[Mesh] OR Argentina[tiab] OR Argentine[tiab] OR Argentinian[tiab] OR Argentinians[tiab] OR Bolivia[tiab] OR Bolivian[tiab] OR Bolivians[tiab] OR Brazil[tiab] OR Brasil[tiab] OR Brazilian[tiab] OR Brazilians[tiab] OR “Central America”[tiab] OR “Central American”[tiab] OR “Central Americans”[tiab] OR Chile[tiab] OR Chilean[tiab] OR Chileans[tiab] OR Colombia[tiab] OR Colombian[tiab] OR Colombians[tiab] OR “Costa Rica”[tiab] OR “Costa Rican”[tiab] OR “Costa Ricans”[tiab] OR Cuba[tiab] OR Cuban[tiab] OR Cubans[tiab] OR "Dominican Republic"[tiab] OR Dominican[tiab] OR Dominicans[tiab] OR Ecuador[tiab] OR Ecuadorian[tiab] OR Ecuadorians[tiab] OR “El Salvador”[tiab] OR Salvadoran[tiab] OR Salvadorans[tiab] OR Salvadorian[tiab] OR Salvadorians[tiab] OR Salvadorean[tiab] OR Salvadoreans[tiab] OR Guatemala[tiab] OR Guatemalan[tiab] OR Guatemalans[tiab] OR Hispanic[tiab] OR Hispanics[tiab] OR Hispano[tiab] OR Hispanos[tiab] OR Honduras[tiab] OR Honduran[tiab] OR Hondurans[tiab] OR "Latin America"[tiab] OR "Latin American"[tiab] OR "Latin Americans"[tiab] OR Latinx[tiab] OR Latina[tiab] OR Latinas[tiab] OR Latino[tiab] OR Latinos[tiab] OR Latine[tiab] OR Latines[tiab] OR Latinus[tiab] OR Mexico[tiab] OR Mexican[tiab] OR Mexicans[tiab] OR Chicana[tiab] OR Chicanas[tiab] OR Chicano[tiab] OR Chicanos[tiab] OR Nicaragua[tiab] OR Nicaraguan[tiab] OR Nicaraguans[tiab] OR Panama[tiab] OR Panamanian[tiab] OR Panamanians[tiab] OR Paraguay[tiab] OR Paraguayan[tiab] OR Paraguayans[tiab] OR Peru[tiab] OR Peruvian[tiab] OR Peruvians[tiab] OR “Puerto Rico”[tiab] OR “Puerto Rican”[tiab] OR “Puerto Ricans”[tiab] OR “South America”[tiab] OR “South American”[tiab] OR “Spanish American”[tiab] OR “Spanish Americans”[tiab] OR Uruguay[tiab] OR Uruguayan[tiab] OR Uruguayans[tiab] OR Venezuela[tiab] OR Venezuelan[tiab] OR Venezuelans[tiab] OR (Spanish[tiab] AND ("Language"[Mesh] OR "Limited English Proficiency"[Mesh] OR English[tiab] OR language[tiab] OR speaking[tiab] OR spoken[tiab] OR speak[tiab] OR speaks[tiab] OR translat*[tiab])) | 430320 |
| 2 | Neoplasms[Mesh] OR Neoplasms[tiab] OR Neoplasm[tiab] OR Neoplasia[tiab] OR Cancer[tiab] OR Cancers[tiab] OR cancerous[tiab] OR Tumor[tiab] OR Tumors[tiab] OR Tumour[tiab] OR Tumours[tiab] OR carcinoma[tiab] OR carcinomas[tiab] OR malignancy[tiab] OR malignancies[tiab] OR malignant[tiab] | 32459 |
| 3 | “Family”[Mesh] OR "Caregivers"[Mesh] OR family[tiab] OR families[tiab] OR caregiver[tiab] OR caregivers[tiab] OR carer[tiab] OR carers[tiab] OR “care giver”[tiab] OR “care givers”[tiab] OR caregiving[tiab] OR “care giving”[tiab] OR relatives[tiab] OR parent[tiab] OR parents[tiab] OR mother[tiab] OR mothers[tiab] OR father[tiab] OR fathers[tiab] OR siblings[tiab] OR sibling[tiab] OR sisters[tiab] OR sister[tiab] OR brothers[tiab] OR brother[tiab] OR grandparent[tiab] OR grandparents[tiab] OR Dyad*[tiab] OR triad*[tiab] OR spouses[tiab] OR spouse[tiab] OR partners[tiab] OR partner[tiab] OR husband[tiab] OR husbands[tiab] OR wife[tiab] OR wives[tiab] OR surrogate[tiab] OR surrogates[tiab] OR stepfamily[tiab] OR stepfamilies[tiab] OR “patient advocate”[tiab] OR “patient advocates”[tiab] OR proxy[tiab] OR “significant other”[tiab] OR “significant others”[tiab] | 3768 |
| 4 | NOT (("Adolescent"[Mesh] OR "Child"[Mesh] OR "Infant"[Mesh]) NOT "Adult"[Mesh]) | 3395 |
| 5 | ("Psychosocial Intervention"[Mesh] OR management[tiab] OR psychoeducation[tiab] OR psychoeducational[tiab] OR psycho-education[tiab] OR psycho-educational[tiab] OR intervention[tiab] OR interventions[tiab] OR program[tiab] OR programs[tiab] OR experiment[tiab] OR experimental[tiab] OR "Clinical Trial"[Publication Type] OR randomized[tiab] OR randomised[tiab] OR randomization[tiab] OR randomisation[tiab] OR placebo[tiab] OR randomly[tiab] OR trial[tiab] OR groups[tiab]) NOT (animals[mh] NOT humans[mh]) | 1513 |

## Database: CINAHL with Full Text (*EBSCOhost*)

| Set # |  | Results |
| --- | --- | --- |
| 1 | MH "Hispanic Americans" OR MH "Central America+" OR MH "Cuba" OR MH "Dominican Republic" OR MH "Latin America" OR MH "Mexico" OR MH "Puerto Rico" OR MH "South America+" OR TI (Argentina OR Argentine OR Argentinian OR Argentinians OR Bolivia OR Bolivian OR Bolivians OR Brazil OR Brasil OR Brazilian OR Brazilians OR “Central America” OR “Central American” OR “Central Americans” OR Chile OR Chilean OR Chileans OR Colombia OR Colombian OR Colombians OR “Costa Rica” OR “Costa Rican” OR “Costa Ricans” OR Cuba OR Cuban OR Cubans OR "Dominican Republic" OR Dominican OR Dominicans OR Ecuador OR Ecuadorian OR Ecuadorians OR “El Salvador” OR Salvadoran OR Salvadorans OR Salvadorian OR Salvadorians OR Salvadorean OR Salvadoreans OR Guatemala OR Guatemalan OR Guatemalans OR Hispanic OR Hispanics OR Hispano OR Hispanos OR Honduras OR Honduran OR Hondurans OR "Latin America" OR "Latin American" OR "Latin Americans" OR Latinx OR Latina OR Latinas OR Latino OR Latinos OR Latine OR Latines OR Latinus OR Mexico OR Mexican OR Mexicans OR Chicana OR Chicanas OR Chicano OR Chicanos OR Nicaragua OR Nicaraguan OR Nicaraguans OR Panama OR Panamanian OR Panamanians OR Paraguay OR Paraguayan OR Paraguayans OR Peru OR Peruvian OR Peruvians OR “Puerto Rico” OR “Puerto Rican” OR “Puerto Ricans” OR “South America” OR “South American” OR “Spanish American” OR “Spanish Americans” OR Uruguay OR Uruguayan OR Uruguayans OR Venezuela OR Venezuelan OR Venezuelans) OR AB (Argentina OR Argentine OR Argentinian OR Argentinians OR Bolivia OR Bolivian OR Bolivians OR Brazil OR Brasil OR Brazilian OR Brazilians OR “Central America” OR “Central American” OR “Central Americans” OR Chile OR Chilean OR Chileans OR Colombia OR Colombian OR Colombians OR “Costa Rica” OR “Costa Rican” OR “Costa Ricans” OR Cuba OR Cuban OR Cubans OR "Dominican Republic" OR Dominican OR Dominicans OR Ecuador OR Ecuadorian OR Ecuadorians OR “El Salvador” OR Salvadoran OR Salvadorans OR Salvadorian OR Salvadorians OR Salvadorean OR Salvadoreans OR Guatemala OR Guatemalan OR Guatemalans OR Hispanic OR Hispanics OR Hispano OR Hispanos OR Honduras OR Honduran OR Hondurans OR "Latin America" OR "Latin American" OR "Latin Americans" OR Latinx OR Latina OR Latinas OR Latino OR Latinos OR Latine OR Latines OR Latinus OR Mexico OR Mexican OR Mexicans OR Chicana OR Chicanas OR Chicano OR Chicanos OR Nicaragua OR Nicaraguan OR Nicaraguans OR Panama OR Panamanian OR Panamanians OR Paraguay OR Paraguayan OR Paraguayans OR Peru OR Peruvian OR Peruvians OR “Puerto Rico” OR “Puerto Rican” OR “Puerto Ricans” OR “South America” OR “South American” OR “Spanish American” OR “Spanish Americans” OR Uruguay OR Uruguayan OR Uruguayans OR Venezuela OR Venezuelan OR Venezuelans) OR (MH "Spanish Language" OR TI (Spanish) OR AB (Spanish) AND (MH "Language+" OR MH "Limited English Proficiency" OR TI ( English OR language OR speaking OR spoken OR speak OR speaks OR translat*) OR AB (English OR language OR speaking OR spoken OR speak OR speaks OR translat*))) | 159986 |
| 2 | MH "Neoplasms+" OR TI (Neoplasms OR Neoplasm OR Neoplasia OR Cancer OR Cancers OR cancerous OR Tumor OR Tumors OR Tumour OR Tumours OR carcinoma OR carcinomas OR malignancy OR malignancies OR malignant) OR AB (Neoplasms OR Neoplasm OR Neoplasia OR Cancer OR Cancers OR cancerous OR Tumor OR Tumors OR Tumour OR Tumours OR carcinoma OR carcinomas OR malignancy OR malignancies OR malignant) | 12876 |
| 3 | MH "Family+" OR MH "Caregivers" OR MH "Significant Other" OR TI (family OR families OR caregiver OR caregivers OR carer OR carers OR “care giver” OR “care givers” OR caregiving OR “care giving” OR relatives OR parent OR parents OR mother OR mothers OR father OR fathers OR siblings OR sibling OR sisters OR sister OR brothers OR brother OR grandparent OR grandparents OR Dyad* OR triad* OR spouses OR spouse OR partners OR partner OR husband OR husbands OR wife OR wives OR surrogate OR surrogates OR stepfamily OR stepfamilies OR “patient advocate” OR “patient advocates” OR proxy OR “significant other” OR “significant others”) OR AB (family OR families OR caregiver OR caregivers OR carer OR carers OR “care giver” OR “care givers” OR caregiving OR “care giving” OR relatives OR parent OR parents OR mother OR mothers OR father OR fathers OR siblings OR sibling OR sisters OR sister OR brothers OR brother OR grandparent OR grandparents OR Dyad* OR triad* OR spouses OR spouse OR partners OR partner OR husband OR husbands OR wife OR wives OR surrogate OR surrogates OR stepfamily OR stepfamilies OR “patient advocate” OR “patient advocates” OR proxy OR “significant other” OR “significant others”) | 2682 |
| 4 | NOT (((MH "Adolescence+" OR MH "Child+" OR MH "Infant+") NOT MH "Adult+"))) | 2428 |
| 5 | (MH "Psychosocial Intervention" OR MH "Clinical Trials+" OR TI (management OR psychoeducation OR psychoeducational OR psycho-education OR psycho-educational OR intervention OR interventions OR program OR programs OR experiment OR experimental OR randomized OR randomised OR randomization OR randomisation OR placebo OR randomly OR trial OR groups) OR AB (management OR psychoeducation OR psychoeducational OR psycho-education OR psycho-educational OR intervention OR interventions OR program OR programs OR experiment OR experimental OR randomized OR randomised OR randomization OR randomisation OR placebo OR randomly OR trial OR groups)) | 1190 |

## Database: APA PsycInfo (*EBSCOhost*)

| Set # |  | Results |
| --- | --- | --- |
| 1 | DE "Latinos/Latinas" OR DE "Mexican Americans" OR TI (Argentina OR Argentine OR Argentinian OR Argentinians OR Bolivia OR Bolivian OR Bolivians OR Brazil OR Brasil OR Brazilian OR Brazilians OR “Central America” OR “Central American” OR “Central Americans” OR Chile OR Chilean OR Chileans OR Colombia OR Colombian OR Colombians OR “Costa Rica” OR “Costa Rican” OR “Costa Ricans” OR Cuba OR Cuban OR Cubans OR "Dominican Republic" OR Dominican OR Dominicans OR Ecuador OR Ecuadorian OR Ecuadorians OR “El Salvador” OR Salvadoran OR Salvadorans OR Salvadorian OR Salvadorians OR Salvadorean OR Salvadoreans OR Guatemala OR Guatemalan OR Guatemalans OR Hispanic OR Hispanics OR Hispano OR Hispanos OR Honduras OR Honduran OR Hondurans OR "Latin America" OR "Latin American" OR "Latin Americans" OR Latinx OR Latina OR Latinas OR Latino OR Latinos OR Latine OR Latines OR Latinus OR Mexico OR Mexican OR Mexicans OR Chicana OR Chicanas OR Chicano OR Chicanos OR Nicaragua OR Nicaraguan OR Nicaraguans OR Panama OR Panamanian OR Panamanians OR Paraguay OR Paraguayan OR Paraguayans OR Peru OR Peruvian OR Peruvians OR “Puerto Rico” OR “Puerto Rican” OR “Puerto Ricans” OR “South America” OR “South American” OR “Spanish American” OR “Spanish Americans” OR Uruguay OR Uruguayan OR Uruguayans OR Venezuela OR Venezuelan OR Venezuelans) OR AB (Argentina OR Argentine OR Argentinian OR Argentinians OR Bolivia OR Bolivian OR Bolivians OR Brazil OR Brasil OR Brazilian OR Brazilians OR “Central America” OR “Central American” OR “Central Americans” OR Chile OR Chilean OR Chileans OR Colombia OR Colombian OR Colombians OR “Costa Rica” OR “Costa Rican” OR “Costa Ricans” OR Cuba OR Cuban OR Cubans OR "Dominican Republic" OR Dominican OR Dominicans OR Ecuador OR Ecuadorian OR Ecuadorians OR “El Salvador” OR Salvadoran OR Salvadorans OR Salvadorian OR Salvadorians OR Salvadorean OR Salvadoreans OR Guatemala OR Guatemalan OR Guatemalans OR Hispanic OR Hispanics OR Hispano OR Hispanos OR Honduras OR Honduran OR Hondurans OR "Latin America" OR "Latin American" OR "Latin Americans" OR Latinx OR Latina OR Latinas OR Latino OR Latinos OR Latine OR Latines OR Latinus OR Mexico OR Mexican OR Mexicans OR Chicana OR Chicanas OR Chicano OR Chicanos OR Nicaragua OR Nicaraguan OR Nicaraguans OR Panama OR Panamanian OR Panamanians OR Paraguay OR Paraguayan OR Paraguayans OR Peru OR Peruvian OR Peruvians OR “Puerto Rico” OR “Puerto Rican” OR “Puerto Ricans” OR “South America” OR “South American” OR “Spanish American” OR “Spanish Americans” OR Uruguay OR Uruguayan OR Uruguayans OR Venezuela OR Venezuelan OR Venezuelans) OR (TI (Spanish) OR AB (Spanish) AND (DE "Language" OR DE "Dialect" OR DE "Figurative Language" OR DE "Foreign Languages" OR DE "Form Classes (Language)" OR DE "Interpreters" OR DE "Monolingualism" OR DE "Multilingualism" OR DE "Native Language" OR DE "Natural Language" OR DE "Phrases" OR DE "Profanity" OR DE "Rhetoric" OR DE "Sentences" OR DE "Sign Language" OR DE "Spelling" OR DE "Vocabulary" OR DE "Written Language" OR DE "Language Proficiency" OR TI ( English OR language OR speaking OR spoken OR speak OR speaks OR translat*) OR AB (English OR language OR speaking OR spoken OR speak OR speaks OR translat*))) | 148483 |
| 2 | DE "Neoplasms" OR DE "Benign Neoplasms" OR DE "Breast Neoplasms" OR DE "Endocrine Neoplasms" OR DE "Leukemias" OR DE "Melanoma" OR DE "Metastasis" OR DE "Nervous System Neoplasms" OR DE "Terminal Cancer" OR TI (Neoplasms OR Neoplasm OR Neoplasia OR Cancer OR Cancers OR cancerous OR Tumor OR Tumors OR Tumour OR Tumours OR carcinoma OR carcinomas OR malignancy OR malignancies OR malignant) OR AB (Neoplasms OR Neoplasm OR Neoplasia OR Cancer OR Cancers OR cancerous OR Tumor OR Tumors OR Tumour OR Tumours OR carcinoma OR carcinomas OR malignancy OR malignancies OR malignant) | 3439 |
| 3 | DE "Family" OR DE "Biological Family" OR DE "Dual Careers" OR DE "Dysfunctional Family" OR DE "Extended Family" OR DE "Family Background" OR DE "Family History" OR DE "Family Members" OR DE "Family of Origin" OR DE "Family Relations" OR DE "Family Resemblance" OR DE "Family Structure" OR DE "Family Work Relationship" OR DE "Interethnic Family" OR DE "Interracial Family" OR DE "Military Families" OR DE "Nepotism" OR DE "Nuclear Family" OR DE "Schizophrenogenic Family" OR DE "Stepfamily" OR DE "Caregivers" OR DE "Couples" OR DE "Same Sex Couples" OR DE "Significant Others" OR TI (family OR families OR caregiver OR caregivers OR carer OR carers OR “care giver” OR “care givers” OR caregiving OR “care giving” OR relatives OR parent OR parents OR mother OR mothers OR father OR fathers OR siblings OR sibling OR sisters OR sister OR brothers OR brother OR grandparent OR grandparents OR Dyad* OR triad* OR spouses OR spouse OR partners OR partner OR husband OR husbands OR wife OR wives OR surrogate OR surrogates OR stepfamily OR stepfamilies OR “patient advocate” OR “patient advocates” OR proxy OR “significant other” OR “significant others”) OR AB (family OR families OR caregiver OR caregivers OR carer OR carers OR “care giver” OR “care givers” OR caregiving OR “care giving” OR relatives OR parent OR parents OR mother OR mothers OR father OR fathers OR siblings OR sibling OR sisters OR sister OR brothers OR brother OR grandparent OR grandparents OR Dyad* OR triad* OR spouses OR spouse OR partners OR partner OR husband OR husbands OR wife OR wives OR surrogate OR surrogates OR stepfamily OR stepfamilies OR “patient advocate” OR “patient advocates” OR proxy OR “significant other” OR “significant others”) | 962 |
| 4 | NOT ( (TI ( child* OR adolescen* OR infan* ) NOT TI (adult*)) ) | 833 |
| 5 | (DE "Psychoeducation" OR DE "Family Intervention" OR DE "Clinical Trials" OR DE "Randomized Controlled Trials" OR TI (management OR psychoeducation OR psychoeducational OR psycho-education OR psycho-educational OR intervention OR interventions OR program OR programs OR experiment OR experimental OR randomized OR randomised OR randomization OR randomisation OR placebo OR randomly OR trial OR groups) OR AB (management OR psychoeducation OR psychoeducational OR psycho-education OR psycho-educational OR intervention OR interventions OR program OR programs OR experiment OR experimental OR randomized OR randomised OR randomization OR randomisation OR placebo OR randomly OR trial OR groups)) | 515 |

## Database: Scopus

| Set # |  | Results |
| --- | --- | --- |
| 1 | TITLE-ABS-KEY ( ( ( argentina OR argentine OR argentinian OR argentinians OR bolivia OR bolivian OR bolivians OR brazil OR brasil OR brazilian OR brazilians OR "Central America" OR "Central American" OR "Central Americans" OR chile OR chilean OR chileans OR colombia OR colombian OR colombians OR "Costa Rica" OR "Costa Rican" OR "Costa Ricans" OR cuba OR cuban OR cubans OR "Dominican Republic" OR dominican OR dominicans OR ecuador OR ecuadorian OR ecuadorians OR "El Salvador" OR salvadoran OR salvadorans OR salvadorian OR salvadorians OR salvadorean OR salvadoreans OR guatemala OR guatemalan OR guatemalans OR hispanic OR hispanics OR hispano OR hispanos OR honduras OR honduran OR hondurans OR "Latin America" OR "Latin American" OR "Latin Americans" OR latinx OR latina OR latinas OR latino OR latinos OR latine OR latines OR latinus OR mexico OR mexican OR mexicans OR chicana OR chicanas OR chicano OR chicanos OR nicaragua OR nicaraguan OR nicaraguans OR panama OR panamanian OR panamanians OR paraguay OR paraguayan OR paraguayans OR peru OR peruvian OR peruvians OR "Puerto Rico" OR "Puerto Rican" OR "Puerto Ricans" OR "South America" OR "South American" OR "Spanish American" OR "Spanish Americans" OR uruguay OR uruguayan OR uruguayans OR venezuela OR venezuelan OR venezuelans ) OR ( spanish AND ( english OR language OR speaking OR spoken OR speak OR speaks OR translat* ) ) ) ) | 1272219 |
| 2 | TITLE-ABS-KEY ( neoplasms OR neoplasm OR neoplasia OR cancer OR cancers OR cancerous OR tumor OR tumors OR tumour OR tumours OR carcinoma OR carcinomas OR malignancy OR malignancies OR malignant ) | 45477 |
| 3 | TITLE-ABS-KEY ( ( family OR families OR caregiver OR caregivers OR carer OR carers OR "care giver" OR "care givers" OR caregiving OR "care giving" OR relatives OR parent OR parents OR mother OR mothers OR father OR fathers OR siblings OR sibling OR sisters OR sister OR brothers OR brother OR grandparent OR grandparents OR dyad* OR triad* OR spouses OR spouse OR partners OR partner OR husband OR husbands OR wife OR wives OR surrogate OR surrogates OR stepfamily OR stepfamilies OR "patient advocate" OR "patient advocates" OR proxy OR "significant other" OR "significant others" ) ) | 7079 |
| 4 | AND NOT (TITLE(child* OR adolescen* OR infan*)) AND NOT (TITLE(adult*)) | 6506 |
| 5 | (management OR psychoeducation OR psychoeducational OR psycho-education OR psycho-educational OR intervention OR interventions OR program OR programs OR experiment OR experimental OR randomized OR randomised OR randomization OR randomisation OR placebo OR randomly OR trial OR groups) | 3868 |

## Database: LILACS

| Set # |  | Results |
| --- | --- | --- |
| 1 | Neoplas$ or cancer$ or tumor$ |  |
| 2 | family or families or caregiver$ or carer$ or "care giver" or "care givers" or caregiving or "care giving" or relatives or parent$ or mother$ or father$ or sibling$ or sister$ or brother$ or grandparent$ or dyad$ or triad$ or spouse$ or partner$ or husband$ or wife or wives or surrogate$ or stepfamily$ or "patient advocate" or "patient advocates" or proxy or "significant other" or "significant others" |  |
| 3 | and not (child$ or adolescen$ or youth) |  |
| 4 | (management or psychoeducation or psychoeducational or psycho-education or psycho-educational or intervention or interventions or program or programs or experiment or experimental or randomized or randomised or randomization or randomisation or placebo or randomly or trial or groups) | 634 |

## Database: SciELO

| Set # |  | Results |
| --- | --- | --- |
| 2 | ( neoplasms OR neoplasm OR neoplasia OR cancer OR cancers OR cancerous OR tumor OR tumors OR tumour OR tumours OR carcinoma OR carcinomas OR malignancy OR malignancies OR malignant ) |  |
| 3 | ( ( family OR families OR caregiver OR caregivers OR carer OR carers OR "care giver" OR "care givers" OR caregiving OR "care giving" OR relatives OR parent OR parents OR mother OR mothers OR father OR fathers OR siblings OR sibling OR sisters OR sister OR brothers OR brother OR grandparent OR grandparents OR dyad* OR triad* OR spouses OR spouse OR partners OR partner OR husband OR husbands OR wife OR wives OR surrogate OR surrogates OR stepfamily OR stepfamilies OR "patient advocate" OR "patient advocates" OR proxy OR "significant other" OR "significant others" ) ) |  |
| 4 | AND NOT (((child OR children OR adolescent OR adolescents OR adolescence OR youth) AND NOT (adult OR adults))) |  |
| 5 | (management OR psychoeducation OR psychoeducational OR psycho-education OR psycho-educational OR intervention OR interventions OR program OR programs OR experiment OR experimental OR randomized OR randomised OR randomization OR randomisation OR placebo OR randomly OR trial OR groups) | 387 |

## Database: ClinicalTrials.gov

| Set # |  | Results |
| --- | --- | --- |
| 1 | Spanish OR Hispanic OR Latino OR Latina OR Latinx) |  |
| 2 | Neoplasms or cancer or tumors |  |
| 3 | (family OR families OR caregiver OR caregivers OR "care giver" OR "care givers" OR caregiving OR "care giving") | 71 |
